# Supplementary material for: Dynamical Quantum Phase Transition and Quasi Particle Excitation
Source: Sci Rep. 2019 Feb 27;9:2871. doi: 10.1038/s41598-019-39595-3 (PMC6393518; doi:10.1038/s41598-019-39595-3)
Supplement: Supplementary file 1 — Supplementary Information [file 41598_2019_39595_MOESM1_ESM.pdf]

Supplemental material to Dynamical Quantum Phase Transition and Quasi Particle Excitation

R. Jafari[1,2,3*]

[1]Department of Physics, Institute for Advanced Studies in Basic Sciences (IASBS), Zanjan 45137-66731, Iran
[2]Beijing Computational Science Research Center, Beijing 100094, China
[3]Department of Physics, University of Gothenburg, SE 412 96 Gothenburg, Sweden
[*]jafari@iasbs.ac.ir, rohollah.jafari@gmail.com

ABSTRACT

0.1 General Compass model

The EQCC ground state $|\psi_0\rangle$ is realized by filling up the negative-energy quasiparticle states, $|\psi_0\rangle = \prod_k \gamma_k^{(1)\dagger}\gamma_k^{(2)\dagger}|0\rangle$, where $|0\rangle$ is the Bogoliubov vacuum annihilated by the γ_k:s[1]. While excited states can be similarly obtained, their construction becomes quite cumbersome within the Bogoliubov-de Gennes formalism. An alternative approach was pioneered by Sun[2]. One here takes off from the observation that the QCC Hamiltonian can be written as a sum of commuting Hamiltonians H_k,

$$H_k = J_k c_k^{A\dagger} c_{-k}^{B\dagger} + L_k c_k^{A\dagger} c_k^{B} + J_{-k} c_{-k}^{A\dagger} c_k^{B\dagger} + L_{-k} c_{-k}^{A\dagger} c_{-k}^{B} + \text{H.c}, \tag{S1}$$

Since H_k conserves the number parity (even or odd number of electrons), it is sufficient to consider the even-parity subspace of the Hilbert space, spanned by

$$\begin{aligned} &|\varphi_{1,k}\rangle = |0\rangle, && |\varphi_{2,k}\rangle = c_k^{A\dagger} c_{-k}^{A\dagger}|0\rangle,\ |\varphi_{3,k}\rangle = c_k^{A\dagger} c_{-k}^{B\dagger}|0\rangle, \\ &|\varphi_{4,k}\rangle = c_{-k}^{A\dagger} c_k^{B\dagger}|0\rangle, && |\varphi_{5,k}\rangle = c_k^{A\dagger} c_{-k}^{B\dagger}|0\rangle,\ |\varphi_{6,k}\rangle = c_k^{A\dagger} c_k^{B\dagger}|0\rangle, \\ &|\varphi_{7,k}\rangle = c_{-k}^{A\dagger} c_{-k}^{B\dagger}|0\rangle, && |\varphi_{8,k}\rangle = c_k^{A\dagger} c_{-k}^{A\dagger} c_k^{B\dagger} c_{-k}^{B\dagger}|0\rangle. \end{aligned} \tag{S2}$$

Given this basis, the eigenstates $|\psi_{m,k}\rangle$ of H_k can be written as $|\psi_{m,k}\rangle = \sum_{j=1}^{8} v_{m,k}^{(j)}|\varphi_{j,k}\rangle$,

0.2 Loschmidt echo

The amplitudes in the mode decomposition of the RP, Eq. (12), depend on the state overlaps $\alpha_{m,k} = |\langle\psi_{m,k}(\theta_2)|\psi_{0,k}(\theta_1)\rangle|^2$ $(m = 0, ..., 7)$ as

$$\begin{aligned} A_{0,k} &= 4\alpha_{0,k}\alpha_{7,k}, \\ B_{0,k} &= 4(\alpha_{2,k} + \alpha_{3,k} + \alpha_{4,k} + \alpha_{5,k})(\alpha_{0,k} + \alpha_{7,k}), \\ A_{1,k} &= 4\alpha_{1,k}\alpha_{6,k}, \\ B_{1,k} &= 4(\alpha_{2,k} + \alpha_{3,k} + \alpha_{4,k} + \alpha_{5,k})(\alpha_{1,k} + \alpha_{6,k}), \\ C_k &= 4(\alpha_{0,k}\alpha_{1,k} + \alpha_{6,k}\alpha_{7,k}), \\ D_k &= 4(\alpha_{0,k}\alpha_{6,k} + \alpha_{1,k}\alpha_{7,k}). \end{aligned}$$

References

1. Jafari, R. Quench dynamics and ground state fidelity of the one-dimensional extended quantum compass model in a transverse field. *J. Phys. A: Math. Theor* **49**, 185004 (2016).
2. Sun, K.-W. & Chen, Q.-H. Quantum phase transition of the one-dimensional transverse-field compass model. *Phys. Rev. B* **80**, 174417 (2009).
